# A Systematic Review of Polygenic Models for Predicting Drug Outcomes

**DOI:** 10.3390/jpm12091394

**Published:** 2022-08-27

**Authors:** Angela Siemens, Spencer J. Anderson, S. Rod Rassekh, Colin J. D. Ross, Bruce C. Carleton

**Affiliations:** 1 Department of Medical Genetics, Faculty of Medicine, University of British Columbia, Vancouver, BC V6H 3N1, Canada; 2 BC Children’s Hospital Research Institute, Vancouver, BC V5Z 4H4, Canada; 3 Division of Translational Therapeutics, Department of Pediatrics, Faculty of Medicine, University of British Columbia, Vancouver, BC V6H 3V4, Canada; 4 Division of Oncology, Hematology and Bone Marrow Transplant, University of British Columbia, Vancouver, BC V6H 3V4, Canada; 5 Faculty of Pharmaceutical Sciences, The University of British Columbia, Vancouver, BC V6T 1Z3, Canada; 6 Pharmaceutical Outcomes Programme, British Columbia Children’s Hospital, Vancouver, BC V5Z 4H4, Canada

**Keywords:** pharmacogenomics, polygenic models, drug outcomes

## Abstract

Polygenic models have emerged as promising prediction tools for the prediction of complex traits. Currently, the majority of polygenic models are developed in the context of predicting disease risk, but polygenic models may also prove useful in predicting drug outcomes. This study sought to understand how polygenic models incorporating pharmacogenetic variants are being used in the prediction of drug outcomes. A systematic review was conducted with the aim of gaining insights into the methods used to construct polygenic models, as well as their performance in drug outcome prediction. The search uncovered 89 papers that incorporated pharmacogenetic variants in the development of polygenic models. It was found that the most common polygenic models were constructed for drug dosing predictions in anticoagulant therapies (*n* = 27). While nearly all studies found a significant association with their polygenic model and the investigated drug outcome (93.3%), less than half (47.2%) compared the performance of the polygenic model against clinical predictors, and even fewer (40.4%) sought to validate model predictions in an independent cohort. Additionally, the heterogeneity of reported performance measures makes the comparison of models across studies challenging. These findings highlight key considerations for future work in developing polygenic models in pharmacogenomic research.

## 1. Introduction

The concept of polygenic inheritance was first introduced in 1918 by R.A. Fisher who showed that continuous traits are passed down through Mendelian inheritance of many genetic variants of small effect [1]. Since then, this polygenic approach to inheritance has been used to study complex human phenotypes [2,3,4,5,6]. Given the small individual effects that each genetic variant contributes to the heritability of complex traits, polygenic scores have emerged as tools to estimate individual probability for these complex phenotypes. Polygenic scores combine the individual effects of several genetic variants into a single score which can be used to assign a probability to any individual representing their genetic predisposition for a phenotype [7,8,9]. As genotyping technologies become increasingly affordable, the excitement surrounding the possibility of generating genome-wide risk scores for various diseases is continually growing [7,10].

Thus far, polygenic scores have primarily been applied in the prediction of disease risk. A highly cited study by Khera et al., published in 2018, developed a polygenic risk score comprised of over 6 million common single nucleotide polymorphisms (SNPs) to predict individual risk of developing coronary artery disease (CAD) [9]. The higher the burden of risk-alleles, the higher an individual’s genetic risk of CAD, and one of the striking discoveries in this study was that patients within the top 8% of the polygenic risk score had a 3-fold increased risk of CAD which is comparable to the risk imparted by rare, monogenic causes of heart disease [9]. The advantage of the polygenic approach is that because it is constructed using common SNPs, it can be applied to many more patients, whereas only a small proportion of the population will carry rare genetic variants. Many other similar polygenic scores have been developed to predict disease risk, and thus, polygenic scores offer the potential to improve genetic screening for disease and are more generalizable to the broader population [11,12].

The polygenic nature of complex traits and disease have become widely accepted, but this has not been translated to the same extent within the field of pharmacogenomics [13]. Innumerable genetic studies have been conducted to explain the interindividual variability in drug-related outcomes such as nonresponse, dosing requirements, and the development of adverse drug reactions (ADRs) [13]. However, many of these early pharmacogenetic studies focused on the monogenic architecture of drug-outcomes where genetic variants of larger effect size were thought of as separate predictors [13]. Relatively few studies have aimed to combine pharmacogenetic variants to improve predictions of these drug outcomes. Perhaps one of the most well-studied multigenic-drug interactions is in warfarin dosing. Warfarin is a widely used oral anticoagulant with a narrow therapeutic window and a high interindividual variability in dosing requirements [14,15]. Early genome-wide association studies of warfarin maintenance dose identified pharmacogenetic variants in *VKORC1* and *CYP2C9* which were strongly associated with warfarin dosing requirements, and genotyping for these variants have been added to the FDA warfarin dosing guidelines [16,17,18]. This highlights the potential utility of pharmacogenetic prediction models comprising multiple genetic loci to guide treatment decisions in clinical practice.

Polygenic scores in pharmacogenomics research were recently reviewed, examining the use of polygenic scores developed from pre-existing genetic studies in disease phenotypes as a predictor of drug outcomes (e.g., schizophrenia-derived polygenic risk score used to predict lurasidone response) [19,20]. However, there has been no review to-date evaluating the use of polygenic models derived specifically from pharmacogenetic variants associated with gene-drug relationships. To this end, a systematic review was conducted to summarize the methods and performance of polygenic models encompassing pharmacogenetic variants in predicting drug outcome phenotypes. In the context of this review, a polygenic model was broadly defined as any model or score encompassing pharmacogenetic variants at more than one genetic locus. In doing so, this review aims to understand the current methods used to develop polygenic models for predicting drug outcomes, as well as the performance of these models in their ability to reliably predict drug outcomes in patients.

## 2. Materials and Methods

The Preferred Reporting Items for Systematic Reviews and Meta-Analysis (PRISMA) guidelines for a systematic review were followed to ensure completeness of the review. A study protocol was written prior to the initiation of the review but was not registered.

### 2.1. Rationale and Scope of Review

This review aimed to summarize the methods and performance of polygenic models encompassing pharmacogenetic variants for predicting drug outcomes. While polygenic models encompassing non-pharmacogenetic variants have been applied to the prediction of drug outcomes, these models were not considered to be true pharmacogenetic models and were not included in the current review. An example of one such article is a 2018 study by Li et al., examining the association between a polygenic risk score derived from schizophrenia risk-alleles and response to lurasidone treatment [20]. While interesting, studies like these were excluded from this review as they draw from polygenic *disease* risk to predict drug outcomes, rather than from *pharmacogenetic* associations with the drug outcome. Articles repurposing phenotype-derived polygenic risk models for drug outcome prediction were similarly excluded. For example, Helmstaedter et al. sought to predict levetiracetam-induced behavioural side-effects using SNPs that predisposed individuals to impulsive, reactive, or aggressive behaviours [21]. As this polygenic model does not incorporate pharmacogenetic variants involved in levetiracetam-induced outcomes, it was not considered a pharmacogenetic model. This is not to say these types of polygenic models are not useful in the prediction of drug outcomes, but simply that they fall outside the scope of the current review. Additionally, these studies were recently reviewed by Johnson et al., so this work sought instead to take a more focused approach in evaluating polygenic models encompassing pharmacogenetic variants only [19].

### 2.2. Search Details

Liberal search criteria were applied in order to capture all relevant articles. For the purposes of this review, a polygenic model was broadly defined as any model or score encompassing pharmacogenetic variants at more than one genetic locus used to stratify patients by genetic risk. Both weighted and unweighted models derived from candidate gene or genome-wide associations were included. Any specific drug-related outcomes were included, such as drug-dosing, therapeutic drug response, or drug-induced adverse effects. Studies that did not investigate pharmacological treatments (e.g., surgical procedures, supplementation, radiation therapy) were excluded. Additionally, studies that did not examine a specific drug (e.g., investigating a chemotherapeutic regimen rather than a specific chemotherapeutic agent) were excluded as it is not possible to associate pharmacogenetic findings to a specific drug-related phenotype. Studies repurposing disease-derived or phenotype-derived polygenic scores to predict drug-related outcomes (e.g., schizophrenia-derived polygenic risk score used to predict lurasidone response) were also excluded, as this was not considered a polygenic model developed using pharmacogenetic variants.

A librarian specializing in medical genetics research was consulted to help construct the search strategy. This was done beginning with MeSH terms, followed by key-words and variations. This strategy was further refined upon review of initial search results in order to ensure all relevant papers were being captured by the search. For example, search terms pertaining to ‘personalized medicine’ were included in the search strategy as some pharmacogenetic studies were filed under this concept within the databases and not necessarily within the ‘pharmacogenetics’ search term. The final search strategy consisted of terms pertaining to “pharmacogenomics”, “pharmacogenetics”, “personalized medicine”, and “polygenic model”. The full search strategy is shown in Figure S1. The search was conducted in MEDLINE and EMBASE using the OVID interface from 1946 to 27 July 2021 for articles that described the development or validation of a polygenic model in human subjects to predict any drug outcomes.

### 2.3. Study Selection

Study screening was performed by two independent reviewers (A.S. and S.A.) in order to minimize bias and retrieve all relevant records pertaining to the research question. Articles were screened for relevancy by their title and abstract, followed by full-text review. To begin the title and abstract screening process, reviewers screened 20 articles for inclusion in full-text review. Conflicts were resolved through discussion between reviewers until a minimum inter-rater reliability of *α* = 0.8 was reached. Articles selected for full review included English language original studies containing a polygenic model used to predict drug outcomes. Conference abstracts, case reports, editorials, notes, meta-analyses, and review articles were not included for full-text review.

### 2.4. Data Extraction

Data extraction was performed by a single reviewer (AS). A standard data extraction sheet was created and piloted on 10 articles, and necessary changes were made to the form before applying it to the full list of included papers. Additional articles were excluded during this process as they were not found to meet all inclusion criteria upon detailed review. Names of the lead authors were extracted, as well as the year of publication. Drugs were classified using the LexiComp database according to their pharmacological category [22]. Details of the drug outcome under investigation were extracted and categorized according to safety, efficacy, or dosing predictions. The method of selecting pharmacogenetic variants for consideration into model development was collected and categorized according to candidate-gene or genome-wide association methods. Details of the model training and validation cohorts were extracted, including population details, as well as number of patients in all study cohorts. Development cohorts were defined as populations used to develop the original score or model, and validation cohorts were defined as any independent population used to test the model’s predictive capabilities. Details of model performance measures were also extracted where available. In cases where the pharmacogenetic prediction model was compared to clinical prediction models, performance measures of the comparison were extracted. If the model was independently validated in an external cohort, predictive performance of the model in this independent population was also extracted. There is currently no risk of bias assessment tool for polygenic model reviews, thus this could not be formally assessed.

### 2.5. Synthesis of Results

Figures, plots, and measures of central tendency used to summarize the included articles were conducted in RStudio Version 1.3.959 for MacOS.

## 3. Results

### 3.1. Overview of Included Articles

**Figure 1:**
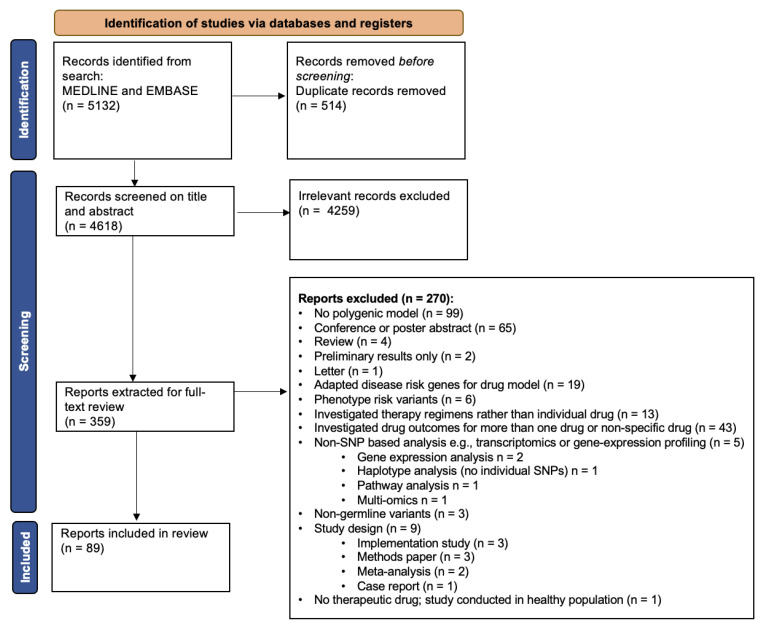
CONSORT flow diagram of articles screened and included in the final review. Flow chart adapted from an example in Page et al. (2021) [23].

The initial literature search conducted in MEDLINE and EMBASE identified 5132 articles. After removal of 514 duplicates, 4618 articles remained for title and abstract screening. From these, 4259 irrelevant studies were excluded by two independent investigators, leaving 359 reports to be extracted for full-text review. Following full-text screening, 100 articles were initially included. During detailed data extraction, an additional 11 reports were excluded as they were found not to include a pharmacogenetic model with multiple genes, leaving 89 papers for inclusion in the systematic review (Figure 1). Full details on included studies can be found in Table S1.

**Figure 2:**
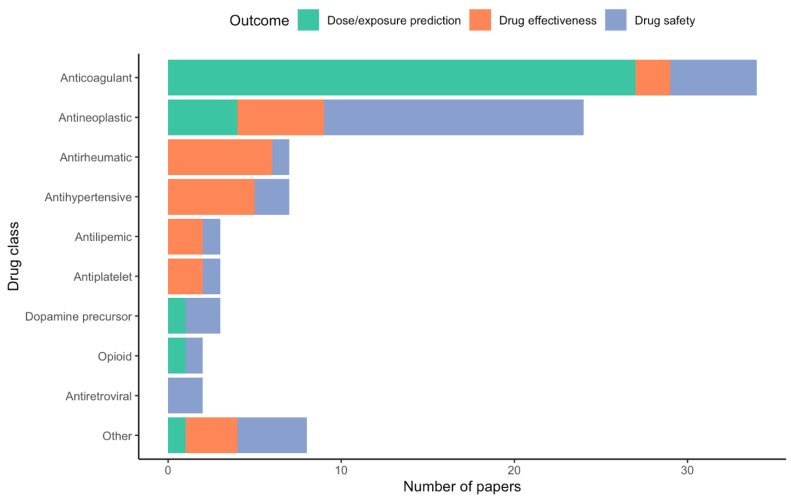
Number of articles included in the review grouped by drug class and investigated drug outcome. The “Other” category is comprised of Sufonylurea antidiabetic (*n* = 1), Angiogenesis inhibitor (*n* = 1), 5-Aminosalicylic acid derivative (*n* = 1), Beta2 agonist (*n* = 1), Antifungal agent (*n* = 1), Antitubercular agent (*n* = 1), Immunosuppressant agent (*n* = 1), and Immune globulin (*n* = 1).

Included papers were published on or before the search date (27 July 2021). The vast majority of drugs for which polygenic predictive models are developed fall under anticoagulants (*n* = 32, 36.0%) or antineoplastic agents (*n* = 22, 24.7%). Of the anticoagulants studied, all were vitamin K antagonists. Drug outcomes under investigation were categorized under three main categories: drug safety (i.e., adverse drug reactions), drug dosing requirements (including drug exposure prediction), and drug effectiveness. Of these, drug dosing requirements was the most common outcome under investigation when developing polygenic prediction models (*n* = 33, 37.1%) [18,24,25,26,27,28,29,30,31,32,33,34,35,36,37,38,39,40,41,42,43,44,45,46,47,48,49,50,51,52,53,54,55], followed by drug safety (*n* = 32, 36.0%) [56,57,58,59,60,61,62,63,64,65,66,67,68,69,70,71,72,73,74,75,76,77,78,79,80,81,82,83,84,85,86,87] and drug effectiveness (*n* = 24, 27.0%) [88,89,90,91,92,93,94,95,96,97,98,99,100,101,102,103,104,105,106,107,108,109,110,111]. The vast majority of studies investigating dosing requirements were conducted in regard to anticoagulant therapy, whereas the majority of drug safety studies were conducted in antineoplastics. A summary of investigated drug outcomes stratified by drug class can be seen in Figure 2.

### 3.2. Method of Gene-Selection for Developing a Polygenic Model Predicting Drug Outcomes

74 of the 89 included studies (83.1%) used a candidate-gene approach when choosing SNPs for inclusion in a multi-pharmacogenetic prediction model. Only 11 studies (12.4%) performed a genome-wide or exome-wide association study to identify pharmacogenetic variants for model development [49,65,69,70,76,86,95,100,102,107,108]. An additional 4 studies (4.5%) aimed to validate a previously published polygenic model [87,109,110,111]. The preference for the candidate-gene approach in these articles may be explained by several factors. Candidate gene analyses are simpler to run and more cost-effective to perform [112,113]. Additionally, sample size constraints remain a challenge in pharmacogenomics research [113]. The median sample size for model development cohorts was 269 patients which would generally be underpowered to accurately estimate allele effect-sizes in a genome-wide study design [7].

Among the candidate-gene approaches, a variety of rationales were used for selection of candidate genes for model development. The most popular method was through literature search to identify variants previously associated with the drug outcome of interest or variants with functional relevance to the drug’s pharmacokinetic or pharmacodynamic pathways. Only two studies explicitly incorporated evidence-threshold criteria in the selection of candidate SNPs. The study by Palles et al. developing a prediction model for capecitabine-induced toxicities used a statistical evidence threshold to select variants associated with the drug outcome in studies of ≥500 patients with an OR/HR of ≥1.5 [75]. Another study by Leusink et al. examining statin-induced cholesterol lowering chose candidate SNPs for model development based on SNPs previously reaching genome-wide significance and replicated in at least one other study for the same drug outcome [104]. For pharmacogenetic models developed using candidate-SNPs, a range of 2 to 60 SNPs were incorporated into the predictive model.

Among the GWA studies, all studies set a *p*-value threshold for choosing SNPs to include in model development. Most studies set this threshold a priori, whereas two studies by Suzuki et al. examining mesalamine allergy and Lanfear et al. examining overall-survival in patients on *β*-blocker therapy used varying *p*-value thresholds to maximize model performance [70,114]. Some studies, like that by Sordillo et al. investigating albuterol response in children with asthma, set a modest *p*-value threshold (*p* < 0.001) but also incorporated functional criteria in SNP-selection by restricting SNPs to those whose predicted functional consequence exceeded 10 on the Combined Annotation Dependent Depletion (CADD) scale [102]. Pharmacogenetic models developed using GWAS included between 5 and 610 SNPs into the predictive pharmacogenetic model.

### 3.3. Overview of Methods Used to Develop Polygenic Predictions Models in Pharmacogenomics

Once pharmacogenetic SNPs were selected for inclusion into a polygenic prediction model, a variety of statistical methods were employed for the development of the models. These include regression-based methods, such as linear, logistic, or Cox proportional hazards regression analyses, and machine learning methods. Machine learning methods varied widely from more common techniques like random forest analyses to newly developed machine learning algorithms. The details of each of the different machine learning methods are beyond the scope of this review, and papers were broadly classified as using regression-based modelling (*n* = 68, 76.4%) or machine learning modelling (*n* = 11, 12.4%). A subset of papers used neither of these, relying instead on pharmacokinetic modelling techniques to create a polygenic prediction model (2 papers, 2.2%) [28,88], Baeysian probability modelling (1 paper, 1.1%) [50], or simply binned patients according to their genotype-category without applying any statistical modelling (7 papers, 7.9%) [42,45,52,59,75,93,105]. No difference was observed between the model development method and the model’s performance (*p* = 0.09). The methods for SNP-selection and modelling technique are summarized in Table 1.

### 3.4. Performance of Polygenic Models in Pharmacogenomics Research

Given the variability in methodologies used to develop polygenic prediction models, it is unsurprising that the same heterogeneity exists for measuring model performance. Methods for assessing model performance included plotting receiver operating characteristic (ROC) curves and calculating area under the curve (AUC) as a measure of model discrimination, R^2^ measures of predictive accuracy, model calibration as measured by the Hosmer-Lemeshow goodness-of-fit test, sensitivity and specificity, positive- and negative-predictive values, mean absolute error, and Pearson correlations (for continuous outcomes only). Some studies did not formally evaluate model performance; rather, patients were binned into risk groups based on polygenic model score and association with the drug outcome was compared between groups. Given the variance in reporting of model performance, direct comparisons could not be drawn between models across different studies.

**Figure 3:**
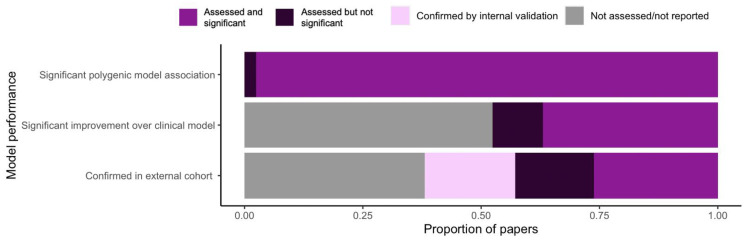
Summary of performance and validation of polygenic models for predicting drug outcomes.

Instead, performance results were interpreted within the context of each individual study by examining (1) whether the polygenic model was successfully associated with the drug outcome of interest, and (2) whether it was able to improve predictions beyond clinical models. Nearly all included studies that developed a model (*n* = 83, 93.3%) identified a significant association between the drug outcome of interest and the pharmacogenetic variants incorporated into the model. However, less than half of these studies (*n* = 42, 47.2%) compared the polygenic model against clinical predictors. Comparisons against clinical predictors are used to demonstrate the added utility of pharmacogenetics beyond clinical factors alone in predicting drug outcomes [115]. Of the studies that did make this comparison, 73.8% showed a significant improvement of the polygenic model over a clinical model. A summary of models reporting significant polygenic associations and improvement over clinical models is shown in Figure 3.

### 3.5. Validating the Performance of Polygenic Models

Over half the included papers (*n* = 56, 63.0%) included some form of model validation in their analysis or were validated in a future study. However, only 36 (40.4%) models were tested in an independent cohort for external validity. As mentioned previously, secondary cohorts of patients treated with the same drug may not be readily available due to sample size constraints. In these cases, some studies (*n* = 16, 18.0%) performed internal validation using cross-validation or internal bootstrap samples to validate their model. Expectedly, internal validation of polygenic prediction models was far more successful than external validation. Where all internally validated models reported successful validation with only a slight reduction in performance, over one third (*n* = 14, 38.9%) of externally validated models did not validate successfully in an independent patient population. Model validation is also summarized in Figure 3.

A very small subset of papers (*n* = 4, 4.5%) was dedicated solely to the independent validation of a previously developed polygenic model. This is in line with the trend in scientific research which has historically favored discovery over replication for publication and explains why most studies aimed to created their own polygenic model rather than validate an existing one [116].

## 4. Discussion

### 4.1. Drug Outcomes Investigated

A wide range of therapeutic classes have been investigated among the included studies in the development of polygenic prediction models (Figure 2). However, the studies reviewed were heavily dominated by anticoagulant (*n* = 32, 36.0%) and antineoplastic (*n* = 22, 24.7%) outcome prediction. Historically, coumarin anticoagulants were extensively studied in the context of pharmacogenomics research due to the widespread prescription of warfarin for the prophylaxis and treatment of venous thromboembolism and other cardiac conditions [117,118,119]. Due to the narrow therapeutic index and high interindividual variability in dosing requirements, ability to predict a patient’s optimal warfarin dose is crucial for avoiding serious adverse drug reactions [120,121]. Prior to any genome-wide studies, researchers and clinicians already suspected that up to 50% of this variability could be explained by patient-specific factors such as age, body mass index, and genetics [14]. Up to one-third of this variability has been associated with variations in the main metabolizer enzyme for coumarin anticoagulants, *CYP2C9*, and the primary drug target, vitamin K epoxide reductase complex I (*VKORC1*) [122]. This prompted the FDA to include pharmacogenetic information on the warfarin drug label, and the International Warfarin Pharmacogenetics Consortium to produce a standard drug-dosing algorithm for warfarin prescription based on genetic information [18,123]. The extensive research on warfarin pharmacogenetics makes it a compelling case study for the polygenic nature of individual drug response, as well as how the use of pharmacogenetic testing can optimize drug outcome predictions.

The pharmacogenetics of anti-cancer therapies have also been extensively investigated. The potent pharmacological agents used to prolong life in cancer can result in severe adverse drug reactions which disproportionately affect cancer patients, with up to 74.3% of hospitalized oncology patients experiencing one or more adverse drug reaction during their stay [124]. As advancements in cancer therapy have improved patient survival, increasing attention has been given to the life-altering and life-threatening adverse effects of chemotherapy [125,126,127]. It is, therefore, unsurprising that the majority of polygenic risk models in cancer therapeutics were developed to predict individual susceptibility to chemotherapy-related adverse drug reactions (Figure 2).

### 4.2. Methods for Polygenic Model Development

In the context of this review, the term ‘polygenic’ was not restricted to the classical definition of “a sum of genome-wide genotypes” [8]. Instead, the term ‘polygenic’ was broadly defined as any pharmacogenetic prediction score or model that encompassed more than one genetic locus in order to also capture pharmacogenetic models not developed from genome-wide studies. Nearly all studies included in this review took a candidate-gene approach when choosing pharmacogenetic SNPs to incorporate into a polygenic prediction model which are widely regarded as inferior to GWA studies due to their hypothesis-driven nature. Linskey and colleagues identified that 94% of genes discovered in pharmacogenomic GWA studies are novel and not previously included in candidate gene studies [128]. This demonstrates the gap in our current understanding of drug pathways and emphasizes the need to shift pharmacogenomic research towards agnostic genome-wide study designs.

The current preference for candidate-gene studies may be explained by the small average sample sizes available in the included articles (median *n* = 269) which would generally be considered underpowered for genome-wide analyses [7]. However, pharmacogenetic variants tend to have larger effects sizes compared to variants associated with other complex traits [129]. While smaller samples may suffice for detection of these larger effect sizes, pharmacogenetic associations of modest effect involved in complex drug pathways may still be missed [113]. This highlights a common challenge within pharmacogenomics research of recruiting sufficiently large samples of uniformly treated patients to perform GWA studies [7,19,113]. This has led many researchers to leverage GWASs derived from large cohorts of related disease phenotypes in the development of polygenic models to predict drug outcome [19]. As mentioned, these studies fell outside the scope of this review as they failed to include pharmacogenetic variants. GWA studies also present additional challenges that may have contributed to the preference for candidate-gene approaches among the included articles. Due to their large scale, GWA studies are often more complex, more time-consuming, and more expensive to run as they require statistical experts familiar with genomic analyses, higher computing power, and specialized genetic analysis software [112].

Nearly all included studies employed regression-based statistical modelling techniques to develop the polygenic prediction models, with only 11 (12.4%) papers using machine learning techniques. Currently, there is not one methodology that produces the best model across all contexts or drug outcomes; rather, it appears that each drug outcome is assessed independently based on the phenotype and study population to determine the most suitable modelling method [115,130]. This is in line with findings from the current review, where no difference was observed between the method used to create the model and the model’s performance (*p* = 0.09).

This suggests that it is perhaps the data used to create the model which has more impact on model performance than the method of model creation [131,132]. For instance, Perini et al., found that a warfarin dosing algorithm developed in a Brazilian population outperformed previous models developed in European populations when applied to Brazilian patients [46]. This is unsurprising given the genetic differences between ancestries. Variant frequency and linkage disequilibrium patterns can vary widely between populations, which often translates to poor performance of polygenic models applied to patients who are different from the input data [133,134,135]. Another study in warfarin dose prediction compared the performance of various models for predicting dosing requirements in children [47]. This study found that the model generated in a pediatric population outperformed those that adapted warfarin dosing models constructed in adults for use in children [47]. This demonstrates that a model performs best within the population for which it was developed, particularly when populations have differing pharmacokinetic profiles [136].

Phenotypic characterization also presents a unique challenge within pharmacogenomic research as many drug outcomes are difficult to measure quantitatively [14]. For example, cisplatin-induced hearing loss is a common adverse drug reaction resulting from cisplatin chemotherapy [137,138,139]. Many pharmacogenomic studies have been conducted to explain the interindividual variability of this adverse outcome, but results are inconsistently replicated [140]. This may be partially explained by the different scales used to grade hearing loss which results in the same patient being assigned into different phenotypic categories depending on the grading criteria used [137,141,142,143,144,145]. Such discrepant phenotyping may result in different polygenic models being constructed depending on the definition of the drug outcome.

There is a wide variety of methods for generating polygenic models in pharmacogenomics research and this diversity continues to increase as different mechanisms arise to overcome challenges in modelling complex drug-related data [115]. This presents a challenge as each drug outcome may have multiple polygenic models with little guidance in choosing the ‘correct’ model to implement clinically. Additionally, polygenic models constructed using more complicated or abstract techniques may face additional barriers toward clinical implementation [131,132,146]. For example, due to the data-driven nature of machine learning methods, learning algorithms are often perceived as a “black box”, manipulating data in unknown ways to generate predictions. Due to the limited interpretability of algorithm results, clinicians and practitioners may have difficulty trusting a model that is not easily explained by current medical evidence [146]. This illustrates a need for data scientists and clinicians to work together in early stages of model development in order to create polygenic prediction models that are clinically useful and interpretable by its intended end-users.

### 4.3. Model Performance

All except for two studies in the current review found significant polygenic associations between the studied polygenic model with the drug outcome of interest. This is in contrast to findings from a review published by Johnson et al. in 2021 where more than half the included studies did not find a significant association between the polygenic risk score and the drug outcome of interest [19]. This difference may be attributed to the fact that variants incorporated into many of the prediction models reviewed by Johnson et al. were disease-related rather than drug-related, and hence did not capture the true pharmacogenetic landscape of the drug outcome under investigation [19]. Articles included in the present review comprised of pharmacogenetic variants previously found to be in direct association with drug outcomes, or with established functional relevance in the drug’s biotransformation pathway. This may explain why the overwhelming majority of studies in this review found a statistically significant association between polygenic models and the drug outcomes. These findings suggest that disease-associated variants cannot always substitute for true pharmacogenetic associations. Pharmacological agents form complex interactions with biological systems through various pharmacokinetic and pharmacodynamic pathways that extend beyond disease mechanisms [147]. Thus, the most robust polygenic models for predicting drug response are those constructed using pharmacogenetic variants. However, these results should be interpreted with caution as studies failing to show a statistically significant association between pharmacogenetic models and investigated drug outcomes may be more likely to remain unpublished [148].

While nearly all studies were able to show a significant genetic association between their polygenic models and drug outcomes, far fewer demonstrated that the inclusion of pharmacogenetic information significantly improved predictions beyond clinical factors alone. Less than half (*n* = 42, 47.2%) the included studies formally compared polygenic versus clinical models for predicting drug outcomes. Of the models that did draw this comparison 73.8% (*n* = 31 out of 42) showed significant improvement over clinical models with the addition of pharmacogenetic factors, suggesting that pharmacogenetics have the potential to improve prediction of drug outcomes over clinical models alone. However, this should also be interpreted with caution due to the low proportion of studies that reported the predictive performance of clinical versus pharmacogenetic models. It is possible that negative results failing to demonstrate improvement of polygenic models over clinical models are less likely to be reported [148]. This may also be due to the lack of established clinical prediction tools against which to compare pharmacogenetic models. Unlike for predicting disease risk, validated clinical prediction tools often do not exist for predicting drug outcomes. Nevertheless, clinical factors have been associated with many drug outcomes of interest. For example, younger-aged children tend to be more at-risk for chemotherapy-induced adverse reactions and body mass index is a well-established predictor for warfarin dosing requirements [149,150,151,152]. Comparisons between these clinical factors and polygenic models are crucial to show clinicians and stakeholders how pharmacogenetics can be used in conjunction with clinical information to result in more effective, individualized therapy [153,154,155,156]. Reporting the extent to which a polygenic model is able to improve (or not) upon clinical predictions where available is likely to play an important role in the implementation of pharmacogenetic testing.

The diversity that exists within model development methods also exists within the reporting of predictive performance. This variability makes comparison and evaluation of polygenic models challenging when trying to decide on the ‘best’ model for use in patients [11]. For instance, the area under the curve (AUC) is the most frequently used metric of a model’s discriminative ability, but it has also been criticized as lacking in other predictive aspects [157]. This has led some authors to instead report metrics of calibration, mean-average-error, or percent variability explained (among many others). Recommendations for reporting practices and guidelines for polygenic model development have been published in the context of disease prediction but are not routinely followed [8,158,159,160]. This inconsistency is apparent in the vast array of performance measures reported among the included studies in this article, and the same trend was observed in a recent review of polygenic risk scores [19]. Improving adherence to standardized reporting guidelines would facilitate comparisons between polygenic prediction models and allow more straightforward evaluation of model performance. Additionally, there are currently no reporting guidelines that are specific to pharmacogenetic polygenic models. Thus, it remains to be seen whether guidelines for disease polygenic models are applicable to pharmacogenetic models, and if so, consensus must be reached on the one(s) to follow in order to facilitate cross-study comparisons.

### 4.4. Model Validation

In order for any prediction model to be implemented, validation of the model must occur in order to demonstrate its predictive performance. In the current review, over one-third of studies (*n* = 33, 37.1%) did not include any obvious form of validation. That is, a polygenic model was fit to the data without testing the validity of genotype-based groupings or predictions. *n* = 16, (18.0%) performed internal validation only using bootstrap or other re-sampling techniques. However, it is widely accepted that it is not sufficient to demonstrate good model performance in the development sample only [161]. In order to demonstrate generalizability, it is essential to confirm that a model maintains good prediction in a different set of individuals than were used for model creation [162]. In this review, a low proportion of articles (*n* = 36, 40.4%) validated the polygenic model in an independent test sample. Only *n* = 4 (4.5%) of the articles were focused solely on conducting an external validation of an existing polygenic prediction model. This low number may be explained by the tendency to preferentially produce novel research rather than attempt to replicate previously published results [116,163,164,165,166,167]. Often, especially until more recent years, publication preference has been given to novel findings [116,163,164,165,166,167]. However, replication of polygenic models for predicting drug outcomes is key to demonstrating their generalizability across patient populations. Generalizability of model predictions has been particularly challenging in the development of polygenic prediction models, with a drop in model performance often observed when applied to a new patient population [135,168,169,170]. This trend is observed in the present study where over one-third of externally validated models failed to predict the drug outcome in an independent cohort (Figure 3.). Part of this challenge reflects a larger bias in genetic research which has primarily been conducted in European populations (Table S1) [113]. As a result, many of these genetic findings are not applicable to populations of different ancestries. Recently, suggestions have been made for reweighting or adjusting models when applied to different populations, but ultimately, there is a need to increase patient-diversity in genetic studies [171]. It has been previously demonstrated that polygenic models developed in more diverse samples have improved generalizability and improved performance when applied to external cohorts of different populations [134]. Thus, improving diversity in pharmacogenetic research is an essential step in creating polygenic models that are widely applicable. Fostering international research collaborations and the formation of large consortia comprised of genetically diverse patients would allow for improved generalizability of pharmacogenetic predictions and more widespread applications of polygenic models.

### 4.5. Study Limitations and Future Directions

This work has several limitations. As discussed previously, the scope of the current study was limited to polygenic models constructed from pharmacogenetic variants only and excluded those derived from disease or phenotype GWAS data. As such, direct comparisons could not be drawn between these different models. Future studies may consider performing a larger-scale review which directly compares these different models, particularly where both are available for the same drug outcomes. This study also excluded polygenic models constructed for multi-drug regimens and thus the results cannot be generalized to drug outcomes resulting from the combined effect of multiple pharmacotherapies. An additional limitation is the exclusion of any non-English language articles as this may have introduced bias into the current study and caused some evidence to be missed. Finally, due to the heterogeneity in reporting of model results, no assessment of publication bias was conducted. As mentioned, negative results are less likely to be reported and thus, the effects of publication bias on the results of the current review cannot be ruled out [148]. This highlights the need to establish clear reporting guidelines for polygenic models predicting drug outcomes, as well as the need to report negative findings to reduce publication bias. Another important consideration in future work is the integration of multiple gene effects (polygenic models) into clinical practice guidelines for pharmacogenetic testing. Currently, clinical practice recommendations for pharmacogenetic testing are predominantly made on a per-gene basis [172,173]. Clear guidelines on clinical interpretation of pharmacogenetic results that combine multiple variants are needed.

In conclusion, the development of polygenic models for predicting drug outcomes is an emerging field with the potential to improve predictions for individual patient response to pharmacological therapy. However, to facilitate advancements in this area of research, consensus is needed surrounding the reporting of model development methods and model performance measures. Additionally, increasing diversity in study populations for polygenic model development can lead to improved generalizability of model predictions and demonstrate clinical utility in a broader group of patients.

## Figures and Tables

**Table 1:** Summary of sample and methods used for developing polygenic prediction models in pharmacogenomics research.

	*n* = 89
**Development cohort size (*n*)**	
Median (range)	269 (37.0, 8726)
**Validation cohort size (*n*)**	
Median (range)	187 (16.0, 14,348)
**Method of SNP-selection for inclusion in polygenic model**	
Candidate-gene	74 (83.1%)
Genome-wide association	11 (12.4%)
Validation of existing polygenic model	4 (4.5%)
**Method for model development**	
Machine Learning	11 (12.4%)
Regression-based method	68 (76.4%)
Other	10 (11.2%)

## Data Availability

Template data collection forms and analytic code used in this study is available from the authors upon request.

## Supplementary Materials

The following supporting information can be downloaded at: https://www.mdpi.com/article/10.3390/jpm12091394/s1, Figure S1: Full search strategy; Table S1: Summary of included articles.
