# Supplementary figures and images for: A Systematic Review of Polygenic Models for Predicting Drug Outcomes
Source: J Pers Med. 2022 Aug 27;12(9):1394. doi: 10.3390/jpm12091394 (PMC9505711; doi:10.3390/jpm12091394)

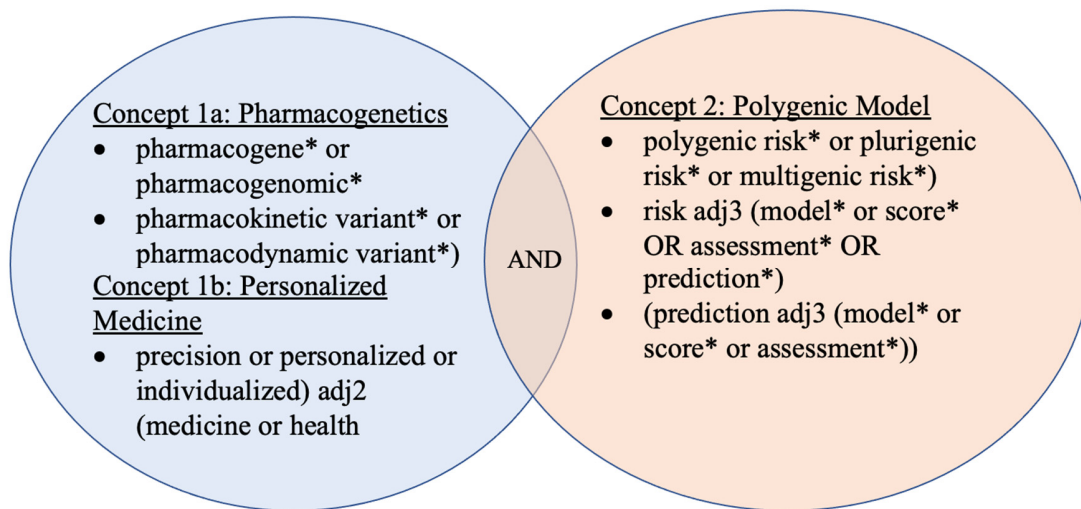

**Figure S1.** Full search strategy. Search was conducted in EMBASE and MEDLINE on July 27, 2021

Supplement: Supplementary file 1 [file jpm-12-01394-s001.zip › Figure S1.pdf]
